# High Prevalence of Clinical Understaging in Early Gastric Cancer: An Analysis of the NCDB

**DOI:** 10.1245/s10434-026-19973-8

**Published:** 2026-07-14

**Authors:** Alexandra Adams, Katherine De La Torre Cisneros, Brijesh Rana, Hayavadhan Thuppal, Mariam F. Eskander, Brett L. Ecker, Miral Sadaria Grandhi, Elizabeth Handorf, Haejin In

**Affiliations:** 1 Division of Surgical Oncology, Rutgers Cancer Institute, New Brunswick, NJ USA; 2 Department of Surgery, Montefiore Medical Center, Bronx, NY USA; 3 Department of Surgery, Northwestern University, Chicago, IL USA; 4 Department of Surgery, Endeavor Health, Evanston, IL USA; 5 Department of Biostatistics and Epidemiology, Rutgers School of Public Health, Piscataway, NJ USA; 6 Department of Health, Behavior, and Policy, Rutgers School of Public Health, Piscataway, NJ USA

**Keywords:** Gastric Cancer, Staging, Outcomes, Clinical Stage, Pathologic Stage

## Abstract

**Background:**

This study aimed to identify the incidence of clinical understaging in early gastric cancer (GC) and to identify variables associated with increased risk of a patient being understaged.

**Methods:**

Clinical stage I (T1/2 N0) GC patients undergoing oncologic resection without neoadjuvant therapy from 2013 to 2020 were identified in the National Cancer Database (NCDB). Patients found to have a higher stage of cancer on final pathologic review were considered understaged. Clinicopathologic predictors of understaging were identified using multivariable logistic regression. The relationship between identified variables and understaging was modeled using logistic regression with natural cubic splines to allow for a flexible, nonlinear analysis. Cox proportional hazards analysis and Kaplan-Meier curves were used to evaluate survival outcomes.

**Results:**

The study identified 4370 clinical stage I GC patients, 36% of whom were initially understaged on clinical staging. Tumor size per millimeter increase (OR, 1.05; 95% confidence interval CI 1.04–1.05; *p* < 0.001), higher grade (moderate: OR, 2.76; 95% CI 1.99–3.84; poor/anaplastic status: OR, 5.99; 95% CI 1.99–3.84, *p* < 0.001), and non-academic treatment facilities (OR, 1.19; 95% CI 1.03–1.38; *p* = 0.017) were associated with increased risk of understaging. Spline analysis showed increased risk of understaging based on tumor size. This was compounded when tumor differentiation was considered, such that a tumor measuring 2.5 cm was associated with 16.9%, 31.8%, or 51.6% likelihood of clinical understaging if the tumor was well, moderately, or poorly differentiated, respectively. Understaging was associated with increased mortality (OR, 2.31; 95% CI 2.10–2.95; *p* < 0.001).

**Conclusions:**

More than one third of clinical stage I GC patients are understaged. Tumor grade and size should be considered at preoperative evaluation of early GC to improve identification of patients at risk for understaging.

**Supplementary Information:**

The online version contains supplementary material available at https://doi.org/10.1245/s10434-026-19973-8.

Gastric cancer (GC) is the fifth leading cause of cancer-related death worldwide.^1^ In the United States, more than 25,000 people have GC diagnosed each year, and nearly half that number of individuals die of the disease annually.^2^

The treatment decisions for patients with GC are primarily guided by the stage of disease at presentation. Upfront surgical resection without neoadjuvant therapy is generally recommended for patients with clinical stage I disease (T1–2, N0).^3^ Conversely, patients with clinical staging of T3 or greater or node-positive disease are offered perioperative chemotherapy before surgery.^4^ As a result, patients initially staged as stage I who undergo upfront surgery but are later found to have more advanced disease on final pathology lose the opportunity to receive neoadjuvant or perioperative chemotherapy.

The benefit of perioperative chemotherapy treatment in the setting of locoregional disease is well-established, with a decrease in tumor size and stage and improved progression-free survival (PFS) and overall survival (OS).^5–7^ The PRODIGY study demonstrated the advantages of neoadjuvant chemotherapy over adjuvant therapy alone. Neoadjuvant therapy improves PFS (hazard ratio [HR], 0.70; 95% confidence interval CI 0.52–0.95; *p* = 0.02), pathologic complete response (pCR) (10.4% vs 0%; *p* < 0.0001), and OS (HR, 0.72; 95% CI 0.54–0.96; *p* = 0.02; 8-year OS rate: 63% vs 55%) compared with upfront surgical treatment followed by adjuvant chemotherapy.^8,9^ Additionally, adjuvant therapy after unexpected upstaging is often delayed, reduced, or omitted due to postoperative complications or prolonged recovery, which has been linked with a worse prognosis.^10,11^

A study using National Cancer Database patients with a diagnosis of clinical early-stage gastric adenocarcinoma (cT1–2N0M0) demonstrated that only 54% of inaccurately staged patients received guideline-concordant care with adjuvant treatment for their locally advanced or metastatic gastric cancer.^12^ When patients are understaged preoperatively, they miss the opportunity for neoadjuvant therapy, which results in suboptimal treatment and worse oncologic outcomes.

Given the clinical implications of understaging, we sought to quantify the frequency of pathologic upstaging of clinical stage I GC patients and identify variables associated with the risk of understaging.

## Methods

### Patient Population

The National Cancer Database (NCDB) is a Commission on Cancer hospital-based cancer registry sponsored by the American College of Surgeons and the American Cancer Society that captures more than 70% of all U.S. patients with a diagnosis of cancer.^13^ This study was approved by the Rutgers University Institutional Review Board (IRB no. CINJ002226) under an exemption determination because the study used de-identified data from a publicly available secondary database.

The NCDB was retrospectively queried for all patients 18 to 90 years old with a diagnosis of gastric adenocarcinoma from 2013 to 2020. Patients were excluded if they had a previous diagnosis of cancer and had not received their diagnosis or initial treatment at the reporting facility. We included patients with gastric adenocarcinoma defined by International Classification of Diseases for Oncology, third edition (ICD-O-3) codes 8140, 8141, 8142, 8143, 8144, 8145, 8146, 8147, 8210, 8211, 8220, 8221, 8231, 8260, 8261, 8262, 8263, 8290, 8310, 8323, 8330, 8440, 8441, 8470, 8471, 8480, 8481, 8490, 8550, 8560, 8562, 8570, and 8572 who had received curative surgical resection. Patients who had received neoadjuvant therapy or had unknown neoadjuvant treatment were excluded. We then limited the patient population to those who had clinical stage I disease (T1N0M0 or T2N0M0) and those who had complete pathologic staging, resulting in the final patient sample used for this analysis.

**Fig. 1:**
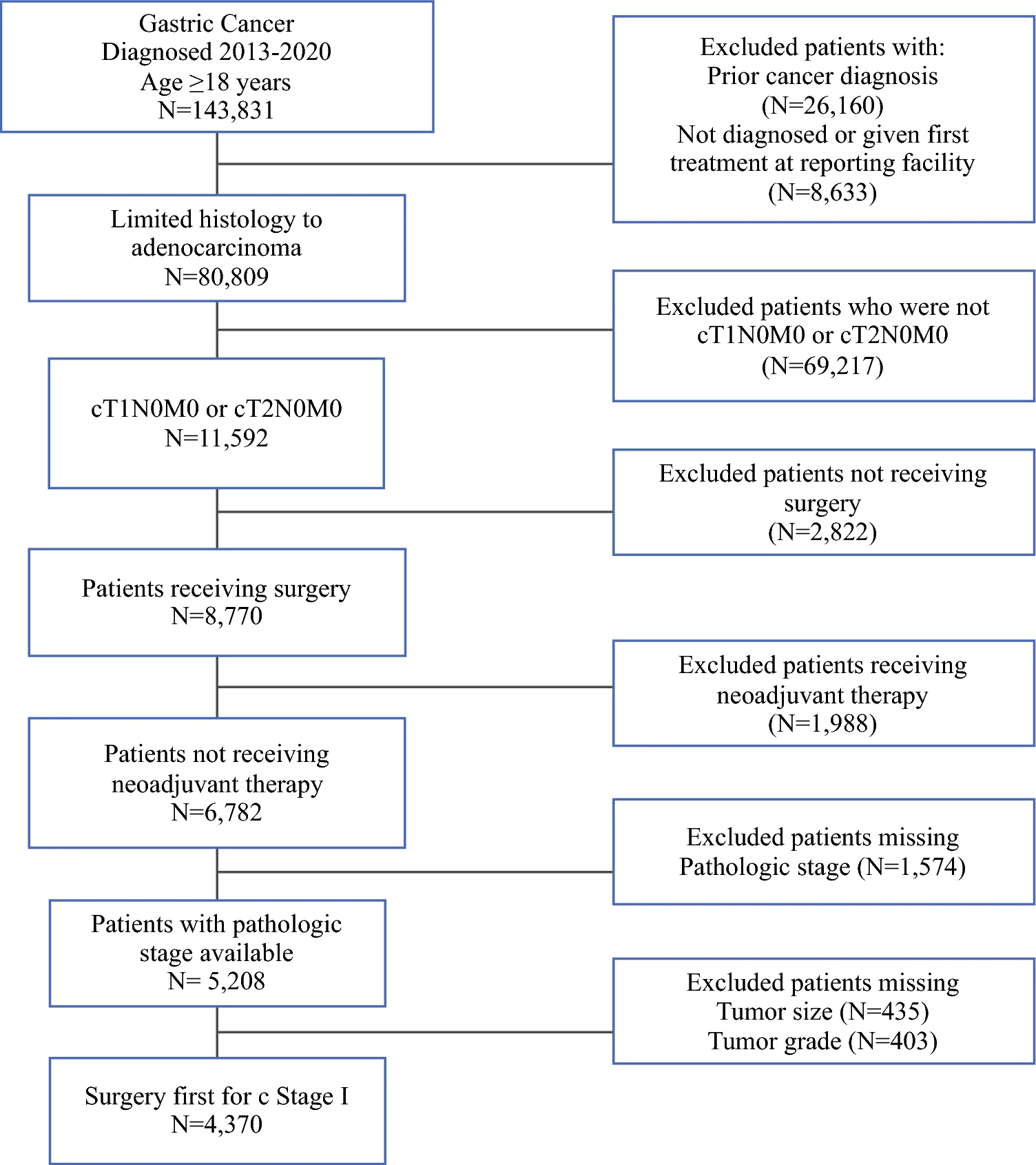
Cohort selection criteria

We identified 6782 clinical stage I (5202 T1N0; 1580 T2N0) gastric adenocarcinoma patients who underwent surgery without neoadjuvant chemotherapy. A number of these patients were missing complete pathologic staging information (*n* = 1574), tumor grade (*n* = 403), and tumor size (*n* = 435), and were removed from analysis. This left a final cohort of 4370 patients with pathologic staging available (Fig. 1).

### Data Collection

Variables on patient demographics, clinicopathologic features, and overall survival were used for analysis. Facilities reporting cases to the NCDB were divided into academic (Academic/Research Program, including NCI-designated comprehensive cancer centers) and non-academic (Community Cancer Program [CCP], Comprehensive CCP, and Integrated Network Cancer Program). Clinicopathologic variables used data derived from the pathologic report.

Tumor histology was categorized using ICD-O-3 codes as signet ring/poorly cohesive (8490, 8142, 8145) or non-signet ring. Tumor location was categorized using ICD-O-3 site codes such as cardia (C160), non-cardia (C161–C166), and other (C168, C169). Patients then were divided into “understaged” and “not understaged” groups based on pathologic stage.

The patients were categorized as understaged if their pathologic stage was greater than their clinical stage (T3 or greater and/or N^+^, M^+^) and not understaged if their pathologic stage was the same or lower compared with the clinical stage. Pathologic staging was defined according to American Joint Commission on Cancer (AJCC) eighth edition. Size of the tumor was analyzed as a continuous variable. Tumor grade was subdivided into three groups: well, moderate, and poor/anaplastic. Carcinoembryonic antigen (CEA) was available for 480 patients (11.0%) and carbohydrate antigen 19-9 (CA19-9) for 190 patients (4.47%). However, due to the large number of patients with missing data, these variables were not included in the final analysis.

### Statistical Analysis

Patient sociodemographic, pathologic, and treatment characteristics were compared between the patients who were understaged and those who were not using descriptive statistics including frequencies and central tendency measurements as well as inferential descriptive data including chi-square test and two-sample *t* test. Absolute and relative frequencies were determined for stage changes from clinical to pathologic stage among the understaged cohort.

Baseline characteristics of included and excluded patients were compared using standardized mean differences (SMDs), with values greater than 0.10 indicating meaningful imbalance.^14^ We found that tumor differentiation and signet ring tumor type were related, so we performed a linear model between tumor differentiation grade and signet ring type to explore their correlation. We also performed a chi-square test to evaluate the independence of the two variables treated as categorical.

Multivariable logistic regression models were used to identify predictors of upstaging using sociodemographic and pathologic variables. Tumor size and grade were found to be predictive, and further analysis was performed to explore size as a predictive variable. We modeled the association between tumor size and understaging using a logistic regression with natural cubic splines to allow for a flexible, nonlinear relationship. Specifically, tumor size was modeled using natural splines with four degrees of freedom, adjusting for age and tumor differentiation grade. This approach captures potential nonlinear effects of tumor size on the odds of understaging without imposing a strict linear assumption^15^ and extends prior NCDB analyses that modeled tumor size categorically.

The Kaplan-Meier method was used to calculate overall survival, comparing patients who were understaged with those who were not. Because understaging is derived from pathologic stage, this analysis is descriptive and was included to illustrate the potential clinical consequences of clinical–pathologic discordance. Cox proportional hazard models were used to examine the effect of understaging on the risk of mortality, considering other variables that influence survival. Both analyses were limited to patients with a diagnosis between 2013 and 2019 to include only patients with complete vital status data (*n* = 4048).

The patients were followed up for a median time of 3.5 years (range, 1 month to 9 years) from their diagnosis date until the date of death or end of the study in 2020. Model 1 was adjusted for age, sex, and race/ethnicity, signet ring presence, tumor differentiation, tumor size, and tumor location. A second model was performed, with further adjustment for chemotherapy and radiation treatment received.

Statistical analysis was performed using SAS software version 9.4 (SAS Institute, Cary, NC, USA) and R version 4.5. Two-sided *p* values lower than 0.05 were considered statistically significant.

## Results

In a final cohort of 4370 patients with clinical stage 1 disease who had pathologic staging available for review, 36% were understaged (*n* =1572). Gender distribution and median age were similar between the two groups. The understaged patients were more likely to be black or Hispanic (black: 12.8% vs 11.8%; Hispanic: 11.6% vs 8.9%; *p* = 0.0343) and more likely to be treated at non-academic versus academic facilities (51.8% vs 44.9%; *p* < 0.0001) than the non-understaged patients.

The understaged patients also were more likely to have poorly differentiated tumors (68.9% vs 42.7%; *p* < 0.0001) and signet ring histology (29.6% vs 19.5%; *p* < 0.0001) than those not understaged. The understaged patients had larger tumors (median: 3.5 cm [interquartile range [IQR] 2.3–5.2 cm] vs 1.8 cm [IQR 1.0–3.0 cm]; *p* < 0.0001) and were less likely to have tumors in the cardia (28.3% vs 37.4%; *p* < 0.0001) than those not understaged. Among the patients who were understaged, 62% received adjuvant chemotherapy, and 32% received adjuvant radiotherapy. In contrast, among those who were not understaged, 4.7% received chemotherapy, and 2.7% received radiotherapy (Table 1).

**Table 1:** Demographic, pathologic, and treatment characteristics of patients understaged compared with those appropriately staged

Variables	Categories	Understaged 36.0% (*n* = 1572) % (*n*)	Not understaged 64.0% (*n* = 2798) % (*n*)	*p* value
Age (years)	<50 50–65 66–75 >75	11.4 (179) 30.6 (481) 29.5 (463) 28.6 (449)	9.4 (262) 29.0 (812) 34.3 (960) 27.3 (764)	0.0051
Sex	Male Female	62.6 (984) 37.4 (588)	63.3 (1772) 36.7 (1026)	0.6287
Race/ethnicity	White Black Hispanic Asian Other	58.0 (912) 12.8 (201) 11.6 (183) 14.3 (224) 3.3 (52)	61.2 (1713) 11.8 (331) 8.9 (250) 14.3 (400) 3.7 (104)	0.0343
Facility type	Academic Non-academic Unknown	45.4 (714) 51.8 (815) 2.8 (43)	52.1 (1459) 44.9 (1257) 2.9 (82)	<0.0001
Histology	Non-signet Signet ring	70.4 (1106) 29.6 (466)	80.5 (2251) 19.5 (547)	<0.0001
Tumor grade	Well Moderate Poor/anaplastic	3.3 (52) 27.8 (437) 68.9 (1083)	16.7 (467) 40.6 (1135) 42.7 (1196)	<0.0001
Tumor size	cm; Median (IQR)	3.5 (2.3–5.2)	1.8 (1.0–3.0)	<0.0001
	<1 >1–2 >2–3 >3–4 >4–6 >6–8 >8–10 >0	3.9 (61) 16.6 (261) 22.8 (359) 19.2 (301) 20.0 (315) 10.2 (160) 3.5 (55) 3.8 (60)	28.8 (805) 30.7 (860) 20.1 (561) 10.3 (289) 7.8 (216) 1.6 (44) 0.5 (13) 0.4 (9)	<0.0001
Tumor location	Non-cardia Cardia Other	58.0 (911) 28.3 (445) 13.7 (216)	51.8 (1449) 37.4 (1046) 10.8 (303)	<0.0001
Received chemotherapy	No Yes Unknown	33.5 (527) 62.0 (974) 4.5 (71)	94.4 (2641) 4.7 (133) 0.9 (24)	<0.0001
Received radiotherapy	No Yes Unknown	65.8 (1034) 32.0 (503) 2.2 (35)	96.7 (2705) 2.7 (75) 0.6 (18)	<0.0001

IQR, interquartile range

We compared the patients included in the analysis with those excluded due to missing pathologic staging (*n* = 1574). Significant imbalances were observed across demographic, tumor, and treatment characteristics (Table S1). The excluded patients were older and more often male and white, with a higher prevalence of cardia tumors, and were less likely to receive chemotherapy or radiotherapy, indicating non‑random missingness.

In this study, CEA and CA19-9 were available for 205 (13.0%) and 105 (6.7%) of the understaged patients and for 275 (9.8%) and 85 (3.0%) of those not understaged, respectively. Among those with available data, median values and distributions for CEA and CA19-9 were similar between the groups (Table S2).

The majority of the patients in the understaged cohort were upstaged from stage I (T1N0; T2N0) to stage II (49.1%), whereas 32.3% were stage III and 3.2% were stage IV on final pathologic staging (Table 2). Of the understaged patients, 19.6% (*n* = 308) were upstaged based on T stage alone, whereas 38.0% (*n* = 598) were upstaged due to node-positive disease. The remaining 39.2% of the understaged patients (*n* = 626) were upstaged due to a combination of T and N upstaging.

**Table 2:** Distribution of post-surgical pathologic stage for understaged cases

AJCC TNM stage 8th edition	% Cohort (*n* = 1572) % (*n*)
Stage IB	15.4
T1N1M0	15.4 (242)
Stage IIA	27.5
T1 N2 M0 T2 N1 M0 T3 N0 M0	5.7 (90) 8.9 (140) 12.9 (203)
Stage IIB	21.6
T1 N3a M0 T2 N2 M0 T3 N1 M0 T4a N0 M0	1.3 (21) 4.1 (65) 10.7 (168) 5.5 (86)
Stage IIIA	18.5
T2 N3a M0 T3 N2 M0 T4a N1 or N2 M0 T4b N0 M0	2.1 (33) 7.4 (116) 8.4 (132) 0.6 (9)
Stage IIIB	10.6
T1 N3b M0 T2 N3b M0 T3 N3a M0 T4a N3a M0 T4b N1 or N2 M0	0.1 (1) 0.4 (6) 4.9 (77) 4.5 (71) 0.7 (11)
Stage IIIC	3.2
T3 N3b M0 T4a N3b M0 T4b N3a or N3b	0.6 (10) 1.8 (29) 0.8 (12)
Stage IV	3.2
M1	3.2 (50)

AJCC, American Joint Committee on Cancer; TNM, tumor-node-metastasis

**Fig. 2:**
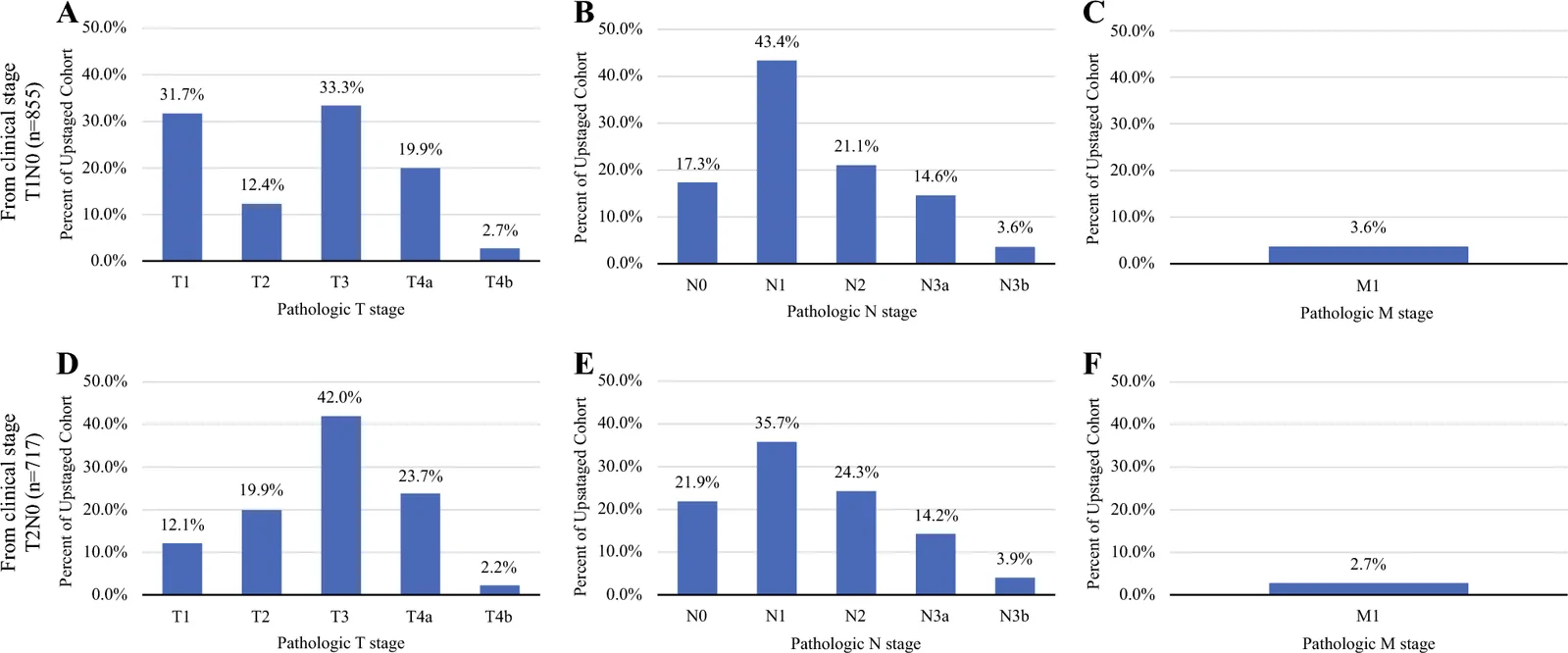
Post-surgical stage distribution of understaged cohort. **A** T stage changes from T1N0. **B** Nodal stage changes from T1N0. **C** Metastasis stage from T1N0. **D** T stage changes from T2N0; **E**. Nodal stage changes from T2N0; **F**. Metastasis stage from T2N0

Notably, 3.2% (*n* = 50) of the patients in the understaged group had pathologic evidence of metastatic disease. Of the patients initially staged as T1N0, 12.4% were upstaged to T2, 33.3% to T3, 22.6% to T4, and 2.7% to T4b (Fig. 2A). In this group, 17.3% remained at N0, 43.4% were upstaged to N1, and 39.3% were upstaged to N2 or higher (Fig. 2B), and 3.6% of these patients were upstaged to M1 (Fig. 2C). Among the patients with an initial clinical stage of T2N0, 12.1% were downstaged to T1, 19.9% remained at T2, 42.0% were upstaged to T3, and 25.9% to T4 or higher (Fig. 2D). In the nodal staging, 21.9% remained at N0, 35.7% were upstaged to N1, and 42.4% were upstaged to N2 or higher (Fig. 2E), and 2.7% of the patients were upstaged to M1 (Fig. 2F).

**Fig. 3:**
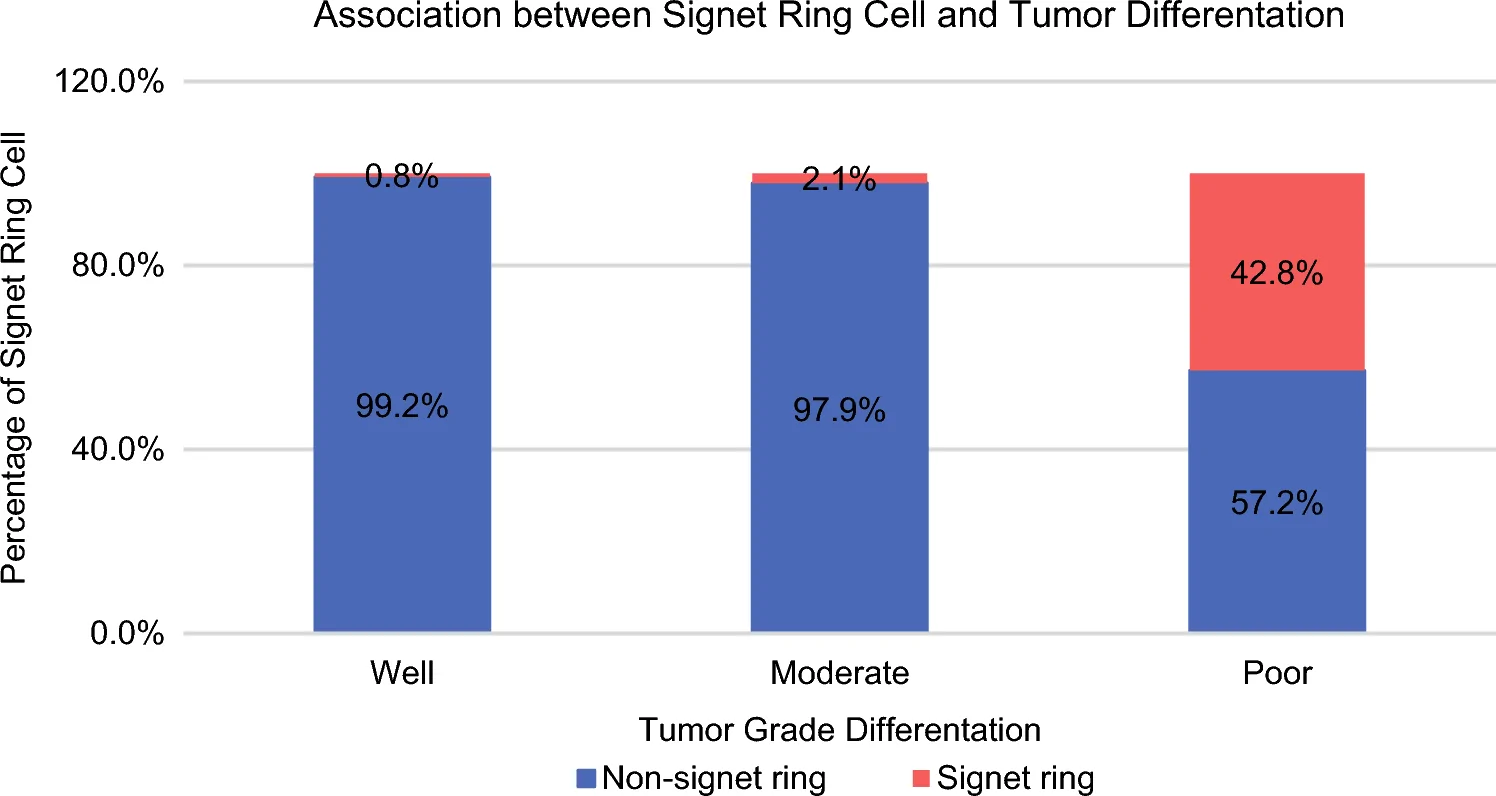
Association between signet ring carcinoma type and tumor grade differentiation

Age, sex, race/ethnicity, tumor size, location, differentiation, and signet ring type were included in the multivariable logistic analysis. The model showed that moderate (OR, 2.76; 95% CI 1.91–3.84) and poor/anaplastic (OR, 5.99; 95% CI 4.31–8.32) tumor differentiation and increasing tumor size (OR, 1.04; 95% CI 1.04–1.05 per mm) were significantly associated with understaging. When the type of facility was added in the model, the patients treated at non-academic facilities had 19% higher odds of understaging than those treated at academic facilities (OR, 1.19; 95% CI 1.03–1.38) (Table 3). On examination of the relationship between signet ring cell type and tumor differentiation, we found that signet ring cell type and differentiation were moderately correlated (*r* = 0.44; *p* < 0.0001). Whereas 42.8% of poorly differentiated tumors were signet ring cell carcinoma, only 2.1% of moderately differentiated and 0.8% of well-differentiated tumors were signet ring cell carcinoma (Fig. 3).

**Table 3:** Multivariable logistic regression on demographic and pathologic factors associated with tumor understaging

Variables	Categories	Model 1		Model 2	
		OR (95% Cl)	*p* value	OR (95% Cl)	*p* value
Age	Risk per year	0.99 (0.99–1.00)	0.051	0.99 (0.98–1.00)	0.011
Sex	Female Male	Reference 1.15 (0.99–1.34)	Reference 0.076	Reference 1.14 (0.98–1.32)	Reference 0.100
Race/ethnicity	White Black Hispanic Asian Other	Reference 0.81 (0.64–1.02) 1.08 (0.85–1.38) 0.93 (0.75–1.15) 0.60 (0.40–0.89)	Reference 0.073 0.539 0.513 0.012	Reference 0.81 (0.64–1.03) 1.08 (0.85–1.38) 0.95 (0.77–1.18) 0.61 (0.41–0.91)	Reference 0.080 0.544 0.635 0.016
Histology	Non-signet ring Signet ring	Reference 0.95 (0.79–1.24)	Reference 0.619	Reference 0.95 (0.79–1.15)	Reference 0.625
Differentiation	Well Moderate Poor/anaplastic	Reference 2.76 (1.99–3.84) 5.99 (4.31–8.32)	Reference <0.0001 <0.0001	Reference 2.90 (2.09–4.01) 6.43 (4.65–8.90)	Reference <0.0001 <0.0001
Tumor size	Risk per millimeter	1.05 (1.04–1.05)	<0.0001	1.05 (1.04–1.05)	<0.0001
Tumor location	Non-cardia Cardia Other	Reference 0.91 (0.76–1.09) 0.86 (0.68–1.08)	Reference 0.258 0.039	Reference 0.91 (0.76–1.08) 0.79 (0.62–1.00	Reference 0.277 0.046
Type of facility	Academic Non-academic Unknown	N/A	N/A	Reference 1.19 (1.03–1.38) 0.77 (0.48–1.22)	Reference 0.017 0.262

OR, odds ratio; CI, confidence interval; N/A, not available

The spline analysis showed an increased probability of understaging based on size. For example, a tumor 1.5 cm in size had a 23.6% likelihood of being understaged, whereas a tumor 3.3 cm in size had a 50.1% risk of being understaged (Fig. S1).

**Fig. 4:**
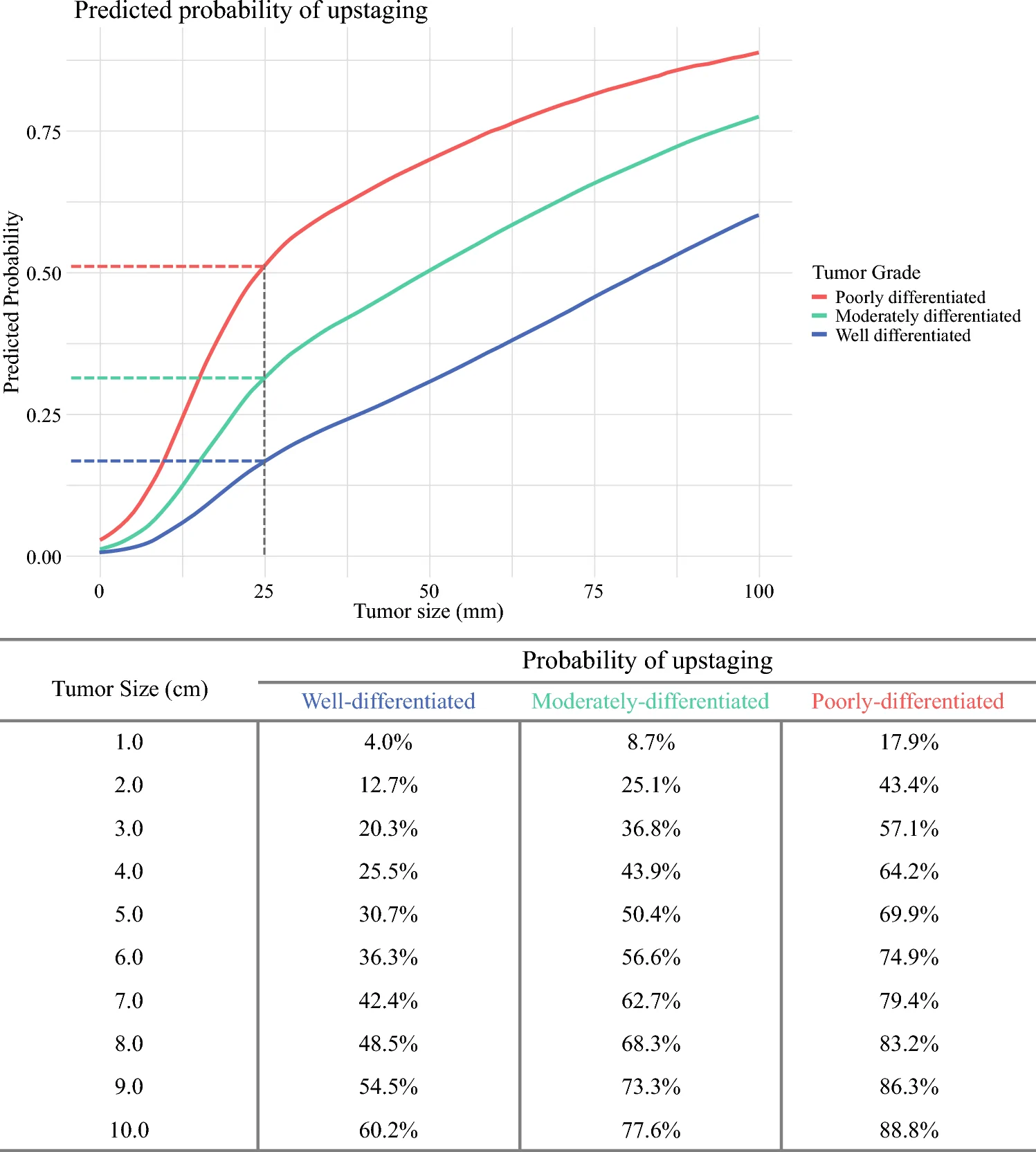
Predicted probability of understaging by tumor size and tumor grade differentiation

Figure 4 shows the interplay of size and differentiation with the probability of understaging. For example, a well-differentiated tumor measuring 2.5 cm had a 16.9% likelihood of being understaged, whereas a moderately differentiated tumor of the same size had a 31.8% likelihood of being understaged. A 2.5-cm tumor that was poorly differentiated had a 51.6% chance of being understaged.

As expected, the patients who were understaged had worse survival, reflecting their true pathologic stage. On survival analysis, the 5-year survival of understaged patients was 50% compared with 77.3% for the patients who were not understaged (*p* < 0.0001; Fig. S1). This descriptive analysis highlights the potential clinical consequences of understaging, while recognizing that pathologic stage remains the primary determinant of prognosis.

In the multivariable Cox-regression analysis, understaging was associated with an increased risk of death when the analysis controlled for tumor and patient demographics (HR, 2.61; 95% CI 2.31–2.95). When treatment variables were included in the analysis, understaging again was associated with elevated risk of mortality (HR, 3.05; 95% CI 2.63–3.52). Similar to factors associated with understaging, poor differentiation (HR, 1.28; 95% CI 1.03–1.59) and larger tumor size (HR, 1.02; 95% CI 1.01–1.03, per centimeter increase) also were associated with worse survival (Table S3).

## Discussion

Accurate clinical staging is critical in guiding treatment selection. For GC, the treatment algorithm for clinical stage I disease differs from higher stages in that patients are offered upfront surgical resection rather than chemotherapy. Unfortunately, the preoperative methods for staging are imprecise. In the current study, we found that more than one third of GC classified as clinical stage I GC was understaged and was ultimately determined to have a higher stage on final pathology. Larger and poorer differentiated tumors had a higher probability of being understaged. These data highlight the inaccuracy of current staging methods in early-stage GC and suggest the need for an accurate report of tumor size and differentiation in addition to standard data to increase the probability of identifying patients at risk of understaging.

Our analysis provides risk for understaging of clinical stage I GC based on tumor size and differentiation. Understaging reflects a more advanced disease than initially recognized clinically, potentially impacting prognosis. If the initial treatment plan is based on an inaccurate lower stage, it precludes the opportunity to treat with neoadjuvant therapy.

These findings support a more nuanced approach to clinical decision-making. Multidisciplinary teams should integrate tumor characteristics, particularly size and grade, together with patient factors such as age, performance status, comorbidities, and nutritional status, among others, when selecting an inital treatment strategy for suspected clinical stage 1 disease. A thoughtful evaluation of risks and benefits is needed to balance the potential for overtreatment against the risk of missing opportunities for optimal care.

Our study found that clinical stage I patients are understaged more than one third of the time. This is in line with prior studies that found rates of nodal positivity upwards of 20% to 40% for cT1N0 GC in this age of endoscopic mucosal resection.^12,16,17^ These data reflect a growing body of work on the inaccuracy of clinical staging in GC.

Accurate clinical staging of GC remains a well-known challenge for health care providers. In a study by Ju et al.^12^ using NCDB, 40% of the patients with a diagnosis of early-stage gastric adenocarcinoma (cT1–2N0M0) between 2009 and 2016 were found to be inaccurately staged. Our analysis of NCDB data from 2013 to 2020 showed a similar rate of inaccurate staging (36%), reflecting only a 4% improvement during nearly a decade.

Beyond updating the timeframe, our study introduced two key refinements to prior studies: (1) modeling tumor size as a continuous variable using restricted cubic splines to capture potential nonlinear associations with understaging and (2) providing an updated assessment of predictors and understaging frequency in a more contemporary cohort. These additions offer both methodologic refinement and contemporary clinical insight into persistent challenges in accurate staging of early gastric cancer. Both computed tomography (CT) and endoscopic ultrasound (EUS), the main methods used for clinical staging, are imperfect in their ability to assign clinical T and N stages. The accuracy of EUS is operator dependent and thus highly variable. One prospective study of more than 1700 patients found that EUS correlated with pathologic T stage for 57% of the patients and N stage for 50% of the patients. Similar to our findings, the same study found that 20% and 25% of the patients were understaged on T stage and N stage, respectively.^18^

Although CT scan protocols and technology have improved, early gastric cancer is often undetectable. Even in more advanced stages, the accuracy of CT remains limited, ranging from 77 to 89% for T stage and N stage. Despite advances such as multidetector CT, substantial limitations persist, with accuracy estimates ranging from 69 to 92%.^19^

To account for differences in quality of staging workup, we modeled risk of understating by type of facility and found that patients treated at academic centers had less risk of understaging. Academic centers often have more specialized resources, advanced imaging, and multidisciplinary teams, potentially resulting in more accurate clinical staging and a lower risk of understaging than non-academic facilities, as displayed by our data.

Our study supports incorporating tumor size and differentiation into clinical decision-making and risk stratification of clinical stage I GC. Routine precise documentation of these characteristics during the initial evaluation is essential to ensure that patients at risk of understaging are accurately identified.

We found tumor size to be the variable most predictive of pathologic upstaging. Data suggest that tumor size is an independent predictor of GC prognosis and strongly associated with lymph node metastasis, particularly in early-stage GC.^17,20–22^ In a retrospective study of patients with tumor invading the submucosa, Gotoda et al.^20^ found that a tumor size greater than 3 cm was an independent risk factor for lymph node metastasis. Using the Surveillance, Epidemiology, and End Results (SEER) database, Chen et al.^17^ found that tumor size smaller than 3.2 cm was an independent predictor of improved 5-year survival in T1 GC patients. Furthermore, they found that including tumor size as a variable in a predictive nomogram improved prediction of overall survival in T1 GC compared with tumor-node-metastasis (TNM) stage alone.

Unfortunately, CT often is unable to detect GC tumors and is not reliable for measuring their size, especially in early GC. Esophagogastroduodenoscopy (EGD) is more accurate at determining tumor size. However, tumor size is not reliably reported by all endoscopists. Given the importance of tumor size in predicting risk of upstaging, our findings support the routine measurement and reporting of tumor size at the time of endoscopy. Although validation in a larger cohort is needed, our data suggest that tumor size could be a useful adjunct in future staging algorithms for early-stage GC.

Tumor differentiation has been associated with prognosis in GC. Poorly differentiated tumors are more likely to present with peritoneal dissemination, lymph node metastasis, and more advanced pathology than well-differentiated tumors.^23,24^ Although correlations have been made between differentiation and signet ring cell presence, studies have shown that differentiation is more indicative of prognosis than the presence of signet ring cells alone.^25^ In fact for early-stage GC, some studies suggest signet ring cell histology may be a favorable prognostic marker.^26,27^ In our study, we did not find that signet ring histology was independently predictive of upstaging in multivariable analysis after accounting for tumor differentiation.

Our study had limitations. It was a retrospective analysis using NCDB, and we were limited in the variables we could analyze based on those collected within this dataset. An important limitation of our analysis was that the NCDB does not separately capture tumor size or differentiation as determined during clinical staging. Therefore, these variables were obtained from the final pathologic report. As such, tumor size and differentiation in our analysis reflect pathologic rather than preoperative clinical assessments. The NCDB also does not capture endoscopic tumor size estimates or biopsy-level histologic grading, precluding evaluation of concordance between preoperative findings and final pathology. Accordingly, these findings should not be interpreted as a directly deployable preoperative prediction model. Nevertheless, our study highlights tumor size and differentiation as key determinants of pathologic understaging, supporting their importance as target variables for future pre-surgical risk stratification approaches aimed at improving patient selection and staging accuracy. Although discrepancies may exist between endoscopic assessment and pathologic measurements, advances in endoscopic imaging and biopsy techniques may enhance the reliability of these factors in preoperative decision-making, posing a question that warrants validation in prospective datasets.

Additionally, NCDB does not capture information on staging methods such as CT or EUS, precluding assessment of their impact on clinical staging accuracy and understaging rates. The database also lacks dates of clinical staging, and surgical dates may not align precisely with initial assessment. Therefore, the effect of time between clinical and pathologic staging on upstaging could not be evaluated.

The NCDB has missing data, and exclusion of patients with incomplete pathologic staging may have introduced downward bias, potentially underestimating the true rate of clinical understaging. In addition, some variables, including CEA and CA19-9, were available only for a subset of patients. Although the utility of these markers in GC have been called into question, they are easily accessible in a clinical setting and could be useful in risk-stratifying early-stage GC. Due to the large amount of missing data, we could not draw meaningful conclusions on these variables. Furthermore, one third of the patients in the original cohort did not have complete pathologic staging, largely reflecting lack of definitive surgical resection or inadequate nodal evaluation, and were excluded from the final analysis, which could affect the generalizability of our results. Because pathologic stage defined the primary outcome, imputation was not feasible and a complete-case analysis was performed. Comparison of included and excluded patients demonstrated meaningful imbalance, suggesting potential selection bias and possible underestimation of the true rate of clinical understaging.

Finally, we acknowledge that recent National Comprehensive Cancer Network (NCCN) guidelines and recent practice-changing clinical trials recommend considering perioperative chemotherapy for T2N0 disease, which may limit the applicability of our definition of who is at risk for upfront surgery due to understaging.^28,29^ However, many T2N0 GC patients still get upfront surgery, and evidence supporting perioperative chemotherapy for this group was limited.^30,31^ During the study period (2013–2020), upfront surgery remained the predominant management approach, with 80.1% of the cT2N0 patients in our cohort proceeding directly to surgical resection. Inclusion of T2N0 patients was therefore intended to reflect real-world staging performance under contemporaneous practice patterns and to evaluate the risk of understaging among patients historically considered appropriate for primary surgery. As treatment standards evolve, the clinical role of preoperative risk stratification may shift toward identifying patients at risk of occult advanced disease who could benefit from multimodality therapy.

## Conclusion

In this study, more than one third of the patients with clinical stage I GC were understaged and subsequently upstaged on post-surgical pathology. Understaging was associated with worse overall survival. Incorporating tumor size and differentiation into the initial evaluation may improve risk stratification for patients with presumed early-stage disease and support more informed treatment selection.

## Supplementary Information

Below is the link to the electronic supplementary material.

Supplementary file1 (DOCX 105 kb)
