# Supplementary material for: High Prevalence of Clinical Understaging in Early Gastric Cancer: An Analysis of the NCDB
Source: Ann Surg Oncol. 2026 Jul 14;33(10):9257–67. doi: 10.1245/s10434-026-19973-8 (PMC13585805; doi:10.1245/s10434-026-19973-8)
Supplement: Supplementary file 1 [file 10434_2026_19973_MOESM1_ESM.docx]

**Supplementary Material**

**Supplemental Table 1.** Characteristics of patients with and without pathological stage information

| **Variables** | **Categories** | **Cohort study (N=4730)** | **Excluded due to no pathological Stage information**  **(N=1674)** | **SMD** |
| --- | --- | --- | --- | --- |
| **Age** | <50  50-65  66-75  >75 | 10.1% (441)  29.6% (1293)  32.6% (1423)  27.8% (1213) | 4.3% (68)  26.2% (412)  32.6% (513)  36.9% (581) | 0.3938 |
| **Sex** | Male  Female | 63.1% (2756)  36.9% (1614) | 70.1% (1104)  29.9% (470) | 0.6215 |
| **Race/Ethnicity** | White  Black  Hispanic  Asian  Other | 60.1% (2625)  12.2% (532)  9.9% (433)  14.3% (624)  3.6% (156) | 77.5% (1219)  7.1% (112)  5.8% (92)  7.2% (114)  2/4% (37) | 0.3818 |
| **Facility type** | Academic  Non-academic  Unknown | 49.7% (2173)  47.4% (2072)  2.9% (125) | 53.3% (839)  45.9% (722)  0.8% (13) | 0.5451 |
| **Histology** | Non-signet  Signet Ring | 76.8% (3357)  23.2% (1013) | 93.1% (1466)  6.9% (108) | 0.6848 |
| **Tumor Grade** | Well  Moderate  Poor/anaplastic  Unknown | 11.9% (519)  36.0% (1572)  52.1% (2279)  0.0% (0) | 13.9% (219)  31.3% (493)  15.8% (249)  39.0% (613) | 0.6141 |
| **Tumor Size** | < 1 cm  >1-2 cm  >2-3 cm  > 3-4 cm  > 4-6 cm  > 6-8 cm  > 8-10 cm  >10 cm | 19.8% (866)  25.7% (1121)  21.1% (920)  13.5% (590)  13.2% (532)  4.7% (204)  1.6% (68)  1.6% (69) | 76.9% (1210)  14.7% (231)  5.4% (85)  1.7% (27)  0.7% (11)  0.6% (9)  0.1% (1)  0.0% (0) | 0.4891 |
| **Tumor Location** | Non-cardia  Cardia  Other | 58.0% (911)  28.3% (445)  13.7% (216) | 29.2% (459)  64.2% (1011)  6.6% (104) | 0.5908 |
| **Received Chemotherapy** | No  Yes  Unknown | 72.5% (3168)  25.3% (1107)  2.2% (95) | 90.6% (1426)  8.5% (134)  0.9% (14) | 0.5946 |
| **Received Radiotherapy** | No  Yes  Unknown | 85.6% (3739)  13.2 (578)  1.2% (53) | 92.3% (1453)  7.0% (110)  0.7% (11) | 0.6421 |
| *Standardized mean difference was calculated to compare the balance between patients with and without missing pathological data. Standardized Difference < 0.10 indicates negligible imbalance; Standardized Difference > 0.20 indicates meaningful differences between groups. | | | | |

**Supplemental Table 2.** Values of CEA (n=480) and CA19-9 (n=190) for understaged and not understaged patients

| **Variables** | **Understaged** | **Not Understaged** | **p-value** |
| --- | --- | --- | --- |
|  | **Median (IQR)** | **Median (IQR)** |  |
| **CEA** | 17 (11-34) | 17 (10-31) | 0.3240 |
| **CA19-9** | 90 (26-230) | 80 (10-196) | 0.3348 |

**Supplementary Figure 1.** Probability of understaging based on tumor size

**Supplementary Figure 2.** Kaplan Meier Survival Curves for clinical stage I Gastric Cancer patients that were understaged vs not understaged, *p<0.001*

**Supplemental Table 3.** Multivariable Cox regression for mortality risk

| **Variables** | **Categories** | **Model 1**  **Hazard Ratio (95% Cl)** | **Model 2**  **Hazard Ratio (95% Cl)** |
| --- | --- | --- | --- |
| **Understaged** | No  Yes | *Reference*  2.61 (2.31-2.95) | *Reference*  3.05 (2.63-3.52) |
| **Age** | Per year increase | 1.046 (1.040-1.051) | 1.043 (1.037-1.049) |
| **Sex** | Female  Male | *Reference*  1.21 (1.07-1.37) | *Reference*  1.21 (1.07-1.38) |
| **Race/Ethnicity** | White  Black  Hispanic  Asian  Other | *Reference*  1.09 (0.92-1.30)  0.83 (0.68-1.03)  0.52 (0.42-0.64)  0.78 (0.54-1.13) | *Reference*  1.09 (0.91-1.29)  0.82 (0.67-1.01)  0.52 (0.42-0.65)  0.79 (0.55-1.15) |
| **Histology** | Non-signet  Signet Ring | *Reference*  1.02 (0.87-1.19) | *Reference*  1.04 (0.89-1.21) |
| **Differentiation** | Well  Moderate  Poor/anaplastic | *Reference*  1.08 (0.87-1.35)  1.27 (1.02-1.58) | *Reference*  1.09 (0.88-1.35)  1.28 (1.03-1.59) |
| **Tumor Size** | Per cm increase | 1.017 (1.011-1.024) | 1.018 (1.012-1.025) |
| **Tumor Location** | Non-cardia  Cardia  Other | *Reference*  1.20 (1.04-1.38)  1.10 (0.92-1.32) | *Reference*  1.19 (1.04-1.37)  1.10 (0.92-1.32) |
| **Chemotherapy** | Yes  No  Unknown | N/A | *Reference*  1.55 (1.27-1.89)  1.96 (1.38-2.78) |
| **Radiation** | Yes  No  Unknown | N/A | *Reference*  0.82 (0.67-1.01)  0.87 (0.56-1.36) |

Abbreviations: N/A, not applicable.
